# Nanosuspension-Based Dissolving Microneedle Arrays for Intradermal Delivery of Curcumin

**DOI:** 10.3390/pharmaceutics11070308

**Published:** 2019-07-02

**Authors:** Sharif Abdelghany, Ismaiel A. Tekko, Lalitkumar Vora, Eneko Larrañeta, Andi Dian Permana, Ryan F. Donnelly

**Affiliations:** 1 Medical Biology Centre, School of Pharmacy, Queen’s University Belfast, 97 Lisburn Road, Belfast BT9 7BL, UK; 2 School of Pharmacy, University of Jordan, Amman 11942, Jordan; 3 Department of Pharmaceutics and Pharmaceutical Technology, Faculty of Pharmacy, Aleppo University, P.O. Box 12212, Aleppo, Syria; 4 Department of Pharmaceutics, Faculty of Pharmacy, Hasanuddin University, Makassar 90234, Indonesia

**Keywords:** curcumin, dissolving microneedles, intradermal, poly(vinylalcohol), nanosuspension

## Abstract

The objective of this study was to evaluate the intradermal delivery of curcumin utilising poly(vinylalcohol) (PVA)-based microneedles loaded with curcumin nanosuspension (CU-NS). Nanoprecipitation was used to formulate the CU-NS which was then incorporated into PVA microneedles arrays consisting of 11 × 11 microneedles of conical shape, measuring 900 µm in height and with 300 µm base diameter. The nanosuspension particle size was 520 ± 40 nm, with a polydispersity of 0.27 ± 0.02 using sodium lauryl sulfate (SLS) as a stabiliser. In vitro dissolution studies in 10% *w/v* Tween 80 showed that the CU-NS dissolved significantly faster than unmodified curcumin powder, with 34% released from the CU-NS, compared to 16% from the curcumin powder after 48 h. The CU-NS-loaded microneedles (CU-MN) were able to withstand a compression force of 32 N for 30 s. Moreover, these microneedles were able to penetrate excised neonatal porcine skin to a depth of 500 µm, dissolved completely in the skin within 60 min. After CU-MN dissolution, the drug diffused from the application site and migrated through the skin layers down to 2300 µm, significantly more than observed with topical application of CU-NS. This suggest that the fabricated microneedles with the incorporated CU-NS could enhance the intradermal delivery of curcumin.

## 1. Introduction

Microneedles are minimally-invasive, painless, drug delivery systems [1]. These micron-sized projection arrays attached to a baseplates are capable of piercing the *stratum corneum*, the principal barrier of the skin, without causing damage to the nerve endings in the dermis. Microneedles can be easily self-administered by the patient [2]. The most widely-used routes of drug administration are the oral and parental routes [3,4]. However, these routes have limitations that include the pain associated with parenteral administration due to impinging on the nerve endings [5] and the low bioavailability due to limited absorption and degradation in the gastrointestinal tract [6]. To overcomes such issues, several types of microneedle arrays have been studied, including hollow microneedles, solid microneedles, coated microneedles, and dissolving microneedles [7]. Rapidly dissolving microneedles (DMN) have attracted considerable interest, since they are composed of water soluble polymers that completely dissolve or degrade in the skin [8]. Understandably, hydrophilic molecules are straightforward to mix with the water-soluble polymers used for DMN preparation [9]. On the contrary, lipophilic compounds do not dissolve in aqueous media; therefore, the incorporation of these compounds in DMN requires the formulation of these drugs in the nanosuspension form to allow homogeneous distribution of the drug in the DMN [10]. The submicron size of nanosuspension particles and hence the greater surface area of the drug particles should result in increased dissolution rate and hence bioavailability after release [11]. However, nanosuspensions are unable to permeate the *stratum corneum*. Therefore, the function of the DMN is to deposit the CU-NS in the viable skin layers. Previously, our group used DMN to enhance skin deposition of a vitamin D nanosuspension [10].

In the present work, curcumin (diferuloylmethane) was incorporated in the form of a nanosuspension into DMN arrays. Curcumin is a yellow pigment present in the spice turmeric (*Curcuma longa*), a member of the ginger family, *Zingiberaceae*, that has been found to possess antioxidant, anti-inflammatory, anticancer, antiviral, and antibacterial activities, as shown by many clinical studies [12]. In addition, recent studies suggest the potential for local effects of curcumin in the management of chronic pain, inflammatory dermatoses, wound healing and skin infections [13]. Previously, it has been shown that topical curcumin is equivalent to orally-administered curcumin in inhibiting growth of cutaneous squamous cell carcinoma, as evidenced by reductions in tumour volume [14]. The aim of this work is to enhance the intradermal delivery of curcumin via DMN. Intradermal administration of nanoformulated drugs could allow sustained local administration, but also prolonged (weeks or months, depending on particle size, drug solubility and potency) systemic absorption, due to presence of the rich dermal microcirculation in the upper dermis. Microneedles create punctures in the *stratum corneum* and deposit their payload in the epidermal and upper dermal layers of the skin. Due to its low solubility in water, curcumin was formulated as a nanosuspension to reduce its size and then incorporated into DMN arrays. This offers a new avenue in enhancing intradermal delivery of curcumin for the treatment of local and systematic diseases, possibly including skin cancers and other skin disorders.

## 2. Materials and Methods

### 2.1. Materials

Poly(vinylalcohol) (PVA), Mw 9–10 kDa, 80% hydrolysed, sodium lauryl sulphate (SLS) and Tween 80 were purchased from Sigma-Aldrich, Poole, Dorset, UK. Optimal cutting temperature (OCT) media was purchased from Scigen Inc, Gardena, CA, USA. Curcumin was purchased from Cambridge Bioscience (Cambridge, UK). Ethanol and acetonitrile were purchased from VWR, Lutterworth, Leicestershire, UK. Water used was double-distilled HPLC grade water (Elga Purelab DV25, Elga LabWater, Lane End, High Wycombe, UK).

### 2.2. CU-NS Preparation

**Figure 1:**
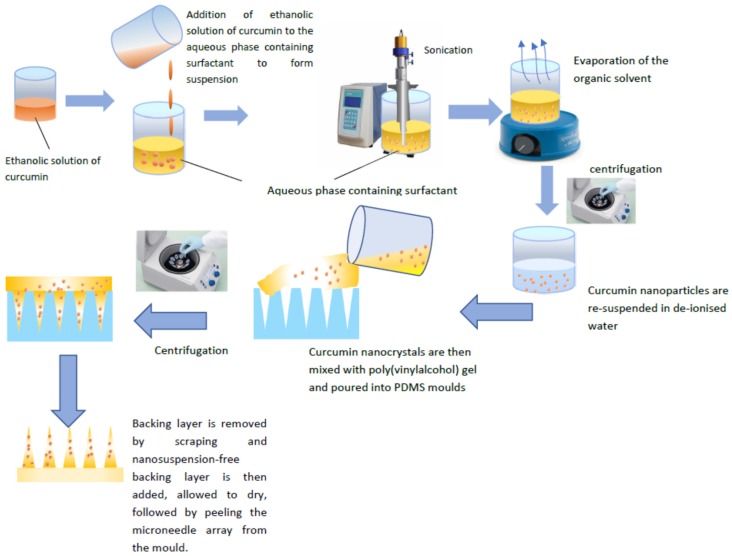
Schematic illustration of curcumin nanosuspension (CU-NS) and dissolving microneedles preparation (CU-MN). CU-MN fabrication used modified nanoprecipitation to first produce the CU-NS, followed by casting the CU-NS polymer mixture on negative poly(dimethylsioloxane) moulds.

CU-NS was prepared using a nanoprecipitation method employing probe sonication, as previously described, with some modifications [15]. Ethanol was used as a solvent and purified water containing stabiliser as antisolvent. Briefly, 2 mL of 2.5 mg/mL curcumin, dissolved in ethanol was added to an aqueous solution composed of 4 mL of 0.2% *w/v* SLS, 10 mL of 0.2% *w/v* SLS, 10 mL of 2% *w/v* PVA, or 10 mL of 0.5% *w/v* Tween 80, under continuous sonication (QS4 system, NanoLab, Waltham, MA, USA). The temperature of the stabiliser solution was maintained at 5 °C ± 3 °C during sonication. The sonication was performed for 5 min at an amplitude of 80% (125 W, 20 KHz) with 10 s pulse on and 5 s pulse off. The nanosuspension was left stirring overnight to evaporate the organic solvent. Eventually, the suspension was centrifuged at 12,800 rpm for 25 min and washed once by spin/resuspension cycle at the same above parameters. Each 1 mL of nanosuspension centrifuged was resuspended in 0.1 mL of deionised water. The process is illustrated in Figure 1.

### 2.3. Microneedle Preparation

Microneedles were prepared using a two-layer centrifugation method, as illustrated in Figure 1. Briefly, 1.5 g of 40% *w*/*w* PVA gel was mixed with 0.6 g of CU-NS and 0.3 g of water using a magnetic stirrer. After that, the mixture of CU-NS and PVA gel was centrifuged at 1000 rpm (Eppendorf^®^ Centrifuge 5804, Merck KGaA, Darmstadt, Germany) for 5 min to remove the air bubbles from the mixture. The resulting mixture was then poured into 11 × 11, poly(dimethylsiloxane) (PDMS) female moulds, with each of the 121 laser-engineered conical holes measuring 900 µm in depth and with 300 µm base diameter. The moulds were then centrifuged at 3500 rpm for 20 min at ambient temperature to allow for the solution to fill the holes. Excess the mixture was then removed by scraping with a spatula and the moulds was kept for 3 h at 37 °C to dry the needles. The aqueous baseplate solution of 20% *w*/*w* PVA was then added to the moulds and centrifuged for 20 min at 2500 rpm. The microneedles were left overnight to dry and then gently peeled from the mould.

### 2.4. CU-NS Characterisation

#### 2.4.1. Particle Size and Zeta Potential

Nanosuspension formulations were characterised for particle size and zeta potential using a NanoBrook Omni Dynamic Light Scattering (DLS) particle sizer and zeta potential analyser (Brookhaven, New York, NY, USA). Measurements were carried out using a monochromatic coherent He-Ne laser light of fixed wavelength (633 nm) at 90° and at room temperature (25 °C), with each sizing determination performed in triplicate and an average particle size expressed as the mean diameter.

#### 2.4.2. Scanning Electron Microscopy

Scanning electron microscopy (SEM) was used to examine the morphology of the particles. Dried nanosuspension powder was sprinkled onto a carbon sticky tape, sputter-coated with 15 nm gold (Quorum Emitech, Kent, UK) and viewed under SEM (Zeiss Ultra Plus scanning electron microscope, Carl Zeiss, Oberkochen, Germany) at an acceleration voltage of 3 kV.

#### 2.4.3. In Vitro Release

In vitro release of curcumin was carried out in 10% *w/v* aqueous Tween 80 at 37 °C to ensure sink conditions [16,17]. An aliquot (10 mg) of freeze-dried CU-NS or curcumin powder was added to the donor compartment (1 mL) and separated from the receptor compartment by 10 KDa cellulose membrane (Snakeskin Dialysis Tubing 10K MWCO, ThermoFisher Scientific, Altrincham, Cheshire, UK). The ratio of volumes between the donor and the receptor compartment was kept at ten to ensure sink condition. At each time point, 1 mL was withdrawn from the receptor compartment and replaced by 1 mL fresh 10% *w/v* Tween 80. Curcumin in the samples was subsequently analysed by high performance liquid chromatography (HPLC).

#### 2.4.4. HPLC Analysis

The analysis of curcumin was conducted using reversed-phase HPLC (Agilent 1200^®^ Binary Pump, Agilent 1200^®^, Standard Autosampler, Agilent 1200^®^ Variable Wavelength Detector; Agilent Technologies UK Ltd., Stockport, UK) with the detection wavelength set at 425 nm. Chromatographic separation was conducted with a Luna C (150 × 4.6 mm 18 (ODS1) with 5 μm packing; Phenomenex, Macclesfield, UK) with 1 mL/min isocratic elution. The mobile phase was a mixture of acetonitrile: 0.3% phosphoric acid (40:60 *v*/*v*).

### 2.5. Microneedle Characterisation

#### 2.5.1. Microneedles Mechanical Strength

The shapes of CU-MN arrays were examined by using a Keyence VHX-700F Digital Microscope (Keyence, Osaka, Japan). A TA.XT-Plus Texture Analyser (Stable Microsystems, Haslemere, UK) was used in compression mode to assess the compression and insertion properties of CU-MN. Heights of CU-MN before compression were first determined using the digital microscope. The curcumin CU-MN arrays were then attached using double-sided adhesive tape to the movable cylindrical probe of the Texture Analyser and pressed by the test station against a flat aluminium block at a rate of 0.5 mm/s for 30 s at a force of 32 N (0.0.264 N/needle) [18]. Pretest and post-test speeds were set at 1 mm/s, and the trigger force was set at 0.049 N. CU-MN heights were measured again, post-compression, using the digital microscope.

#### 2.5.2. CU-MN Drug Content

The drug content of the CU-MN was achieved by carefully cutting the microneedles from the baseplate with a scalpel and collecting them in 1.5 mL Eppendorf^®^ tubes. The microneedles were then dissolved in the mobile phase and analysed using HPLC.

#### 2.5.3. Microneedles Insertion Studies

To determine insertion properties of the CU-MN arrays, Parafilm M^®^ (Bemis Company Inc., Soignies, Belgium), an artificial flexible thermoplastic sheet made of olefin-type material, was used. Parafilm M^®^ has been previously validated as a skin simulant for insertion of microneedles [19]. Heights of the CU-MN arrays were measured microscopically prior to the test. The Parafilm M^®^ sheet was folded into an eight-layer film (≈1 mm thickness). Following attachment of the CU-MN array to the movable probe of the Texture Analyser, the probe was lowered onto the folded Parafilm M^®^ at a speed of 1.19 mm/s until the required force of 32 N was exerted and held for 30 s. The CU-MN were then removed from the Parafilm M^®^ sheet after insertion. The Parafilm M^®^ sheet was unfolded and the number of holes in each layer counted. The retrieved DMN had their heights evaluated using a Leica EZ4 D digital microscope (Leica Microsystems, Wetzlar, Germany).

#### 2.5.4. Microneedles Dissolution in Skin

**Figure 2:**
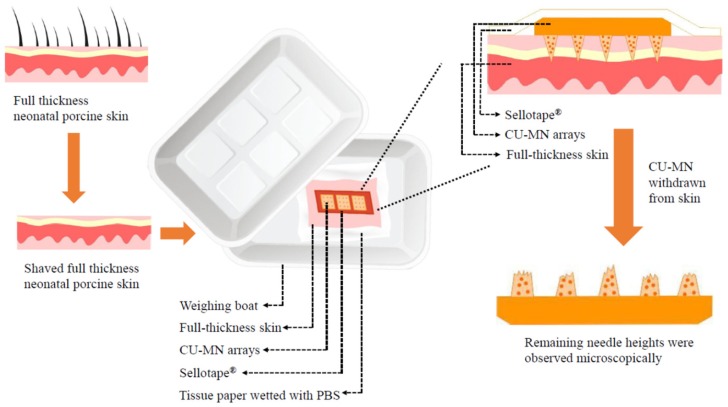
Schematic representation of the set-up used to study dissolution of curcumin nanosuspension-loaded microneedle arrays (CU-MN) in full-thickness neonatal porcine skin in vitro.

Neonatal porcine skin was used as a skin model, due to its similarities to human skin in terms of general structure, thickness, hair density, pigmentation, collagen and lipid composition [20]. Neonatal porcine skin samples were obtained from stillborn piglets and immediately (<24 h after birth) excised. Skin samples were stored in sealed Petri dishes at −20 °C until use. Prior to use, skin samples were shaved and equilibrated in phosphate buffered saline (PBS), pH 7.4, for 15 min prior to use. One section of full thickness neonatal porcine skin was placed, dermal side facing downwards, onto a piece of tissue paper wetted with PBS in a weighing boat. CU-MN arrays were adhered to a piece of Sellotape^®^ and manually applied to the skin. In order to prevent the skin from drying out, another weighing boat was used as a cover, with the join sealed using Sellotape^®^. At predefined time points, CU-MN were withdrawn from the skin and their heights measured using the digital microscope (Figure 2).

#### 2.5.5. Optical Coherence Tomography

Optical coherence tomography (OCT) was employed to visualise the cross-sectional insertion of CU-MN in neonatal porcine skin, as previously described [21]. Briefly, the skin surface was dried using tissue paper and placed dermis side down on a dental wax sheet. CU-MN arrays were then inserted using the Texture Analyser, with a force of 32 N again applied for 30 s. OCT images were then recorded using an EX1301 OCT Microscope (Michelson Diagnostics Ltd., Kent, UK).

#### 2.5.6. Ex Vivo Drug Deposition in Skin

The distribution of curcumin in full thickness neonatal porcine skin was investigated ex vivo using a cryostatic microtome, as described previously [22]. The full thickness neonatal porcine skin was excised and treated as above. CU-MN arrays were then inserted into the skin using manual pressure for 30 s applied to the microneedle baseplate. A cylindrical 10 g stainless steel weight was placed on top of the CU-MN array to prevent microneedles expulsion and the tissue paper was frequently wetted with PBS to avoid skin drying out. At each predefined time point, the microneedles were removed, the skin was placed on dental wax and 1 cm^2^ of skin was excised using a cork borer. The skin was then fully immersed in OCT media and frozen by immersion in liquid nitrogen. The frozen skin was then sliced horizontally in layers of 200 µm using a cryostatic microtome (Leica CM1900-1-1 cryostatic microtome, Leica Microsystems, Nussloch, Germany). Prior to quantification of each layer’s drug content, skin samples were mixed with 1 mL of acetonitrile, sonicated for 4 h to extract curcumin and were then centrifuged for 30 min at 12,000× *g* using an Eppendorf Mini-spin centrifuge (Eppendorf UK Ltd., Stevenage, UK). All samples were analysed using the developed reverse-phase HPLC method. Drug distribution resulting from control CU-NS formulations was studied in the same manner except, instead of inserting a CU-MN array, formulations were placed on top of the skin, followed by the stainless-steel weight, for consistency.

### 2.6. Statistical Analysis

The results are presented as means ± standard deviation (SD) of the mean. Statistical comparison between CU-MN and CU-NS, in terms of curcumin permeation, was made using GraphPad Prism software (ver. 7; GraphPad, Inc. San Diego, CA, USA). A two-tailed Student’s *t*-test was used to compare different pairs of data. One-way analysis of variance (ANOVA) was used in the skin disposition study to compare the effects of different formulations and/or times of incubation. Rejection of the null hypothesis was considered when *p* < 0.05.

## 3. Results

### 3.1. Nanosuspension Characterisation

**Figure 3:**
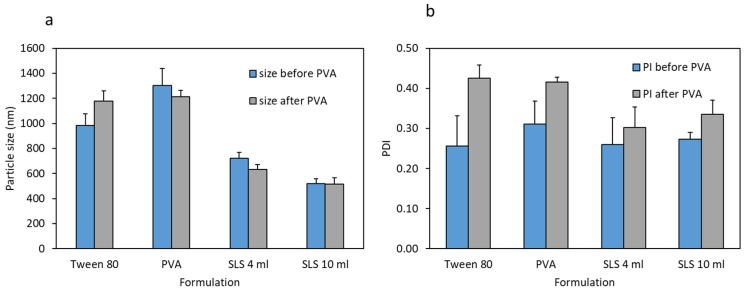
(**a**) Particle size of CU-NS for different surfactants in aqueous solutions before and after mixing with poly(vinylalcohol) (PVA) solutions; (**b**) Polydispersity of CU-NS for different surfactants in aqueous solutions before and after mixing with PVA. Means + S.D., *n* = 3.

Figure 3 shows the particle sizes and polydispersity indices (PI) of the various CU-NS formulations before and after mixing with PVA solutions. The results show that the lowest particle size was achieved with SLS. Accordingly, since the desire was to enhance release rate, this formula was used for further investigations. The zeta potential for the SLS formulations were in the neutral range. This indicates that the surfactant is not adsorbed in significant amounts to the CU-NS surfaces following the washing step. Aqueous SLS formulations (both 4 mL and 10 mL) showed PI values lower than 0.35. Finally, when these particles were combined with PVA polymer solution, the resulting particles showed no significant increase in their size or PI. Moreover, reducing the volume of the aqueous solution from 10 mL to 4 mL resulted in a significant increase in the particle size, as shown in Figure 3a,b.

Table 1 shows the Zeta potential of the CU-NS before and after mixing with the PVA solutions. The Zeta potential was shown to be slightly negative before mixing with PVA. No significant difference was shown in either case for formulations before or after mixing with PVA solutions (*p* < 0.05).

**Figure 4:**
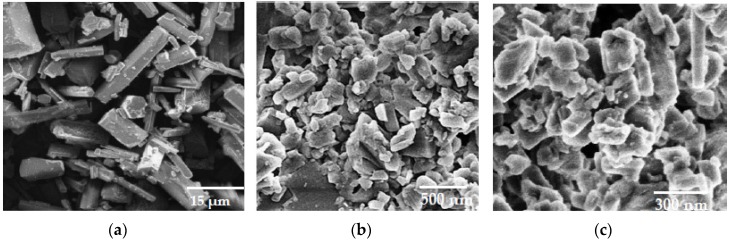
(**a**) Scanning electron microscopy (SEM) images of curcumin (**a**) Scanning electron microscopy (SEM) images of curcumin powder; (**b**) SEM images of CU-NS for the 10 mL/0.2% SLS formula before mixing with PVA solution; (**c**) SEM images of CU-NS after mixing with PVA solution.

The SEM images of curcumin powder showed crystalline particles greater than 15 µm in diameter (Figure 4a). However, CU-NS, before and after mixing with PVA solutions, showed particle sizes that corroborate the results obtained from dynamic light scattering. The crystalline nature of curcumin appeared to be preserved (Figure 4b,c).

**Figure 5:**
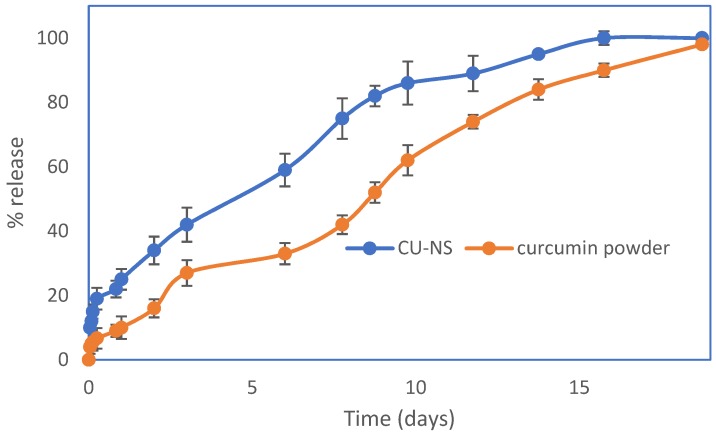
*In vitro* dissolution/release of curcumin powder and CU-NS in 10% *w/v* aqueous Tween 80 media at 37 °C. Means ± S.D., *n* = 3.

Studying the in vitro dissolution of curcumin powder and the optimised CU-NS showed that the nanosuspension provide faster dissolution compared to curcumin powder (Figure 5). Complete dissolution was achieved for curcumin powder after 18 days and after 14 days for CU-NS.

### 3.2. Microneedle Characterisation

#### 3.2.1. In Vitro Characterisation of Microneedles

**Figure 6:**
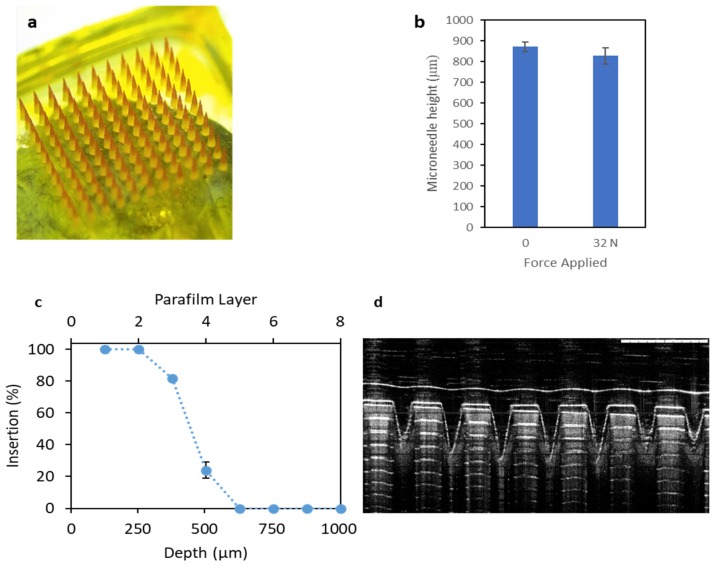
(**a**) Panoramic light microscope image of CU-MN; (**b**) CU-MN height before and after application of 32 N force for 30 s (means + S.D., *n* = 3); (**c**) insertion depth and number of Parafilm M^®^ layers perforated during CU-MN application (means ± S.D., *n* = 3) and (**d**) optimal cutting temperature (OCT) image of perforated Parafilm M^®^ after CU-MN application (Scale bar = 1 mm).

Following optimisation of the CU-NS formulation, microneedle arrays were prepared using PVA polymer solution combined with CU-NS. The arrays were properly formed, as can be seen in Figure 6a. These images showed that the needle tips presented a two-layered conical structure. Each CU-MN array showed a needle height of around 900 μm and a base diameter of 300 µm. The CU-NS appeared to be more concentrated at the tips of the microneedles, which were relatively sharp. The microneedle height before and after applying a 32 N force for 30 s were not significantly different (*p* = 0.18), as shown in Figure 6b. After incorporating the 10 mL SLS CU-NS formula into PVA-based microneedles, the amount of curcumin in the microneedles array was shown to be approximately 10.9 ± 1.1 µg/array.

As shown in Figure 6c, the microneedles were able to penetrate between three and four layers of Parafilm M^®^. This can be corroborated using the OCT penetration study (Figure 6d). Since each layer is approximately 126 µm thick, it is reasonable to assert that the CU-MN can penetrate to depths between 280–500 µm (42–56% of the microneedle length).

#### 3.2.2. Performance of Microneedles in Ex Vivo Neonatal Porcine Skin

**Figure 7:**
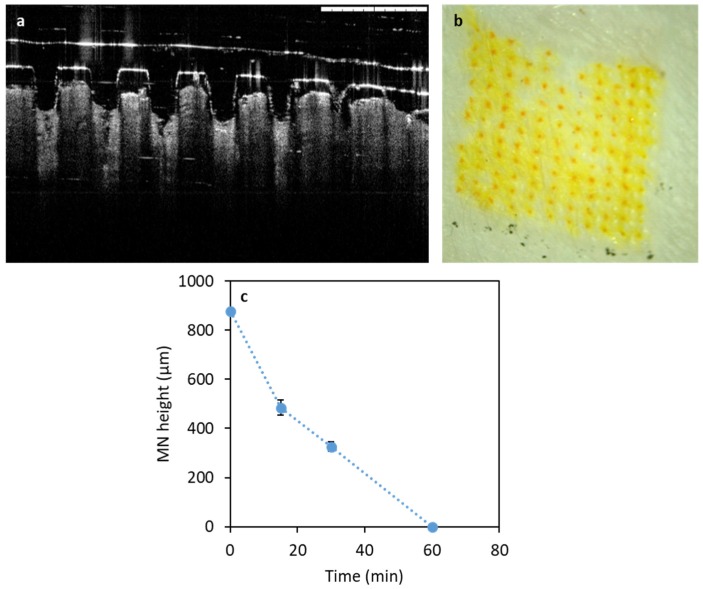
(**a**) OCT of CU-NS MN in ex vivo neonatal porcine skin (Scale bar = 1 mm); (**b**) Light microscope of CU-MN treated neonatal porcine skin and (**c**) CU-MN height versus time after insertion into porcine skin. Means ± S.D., *n* = 3.

CU-MN microneedles were confirmed to penetrate neonatal porcine skin, as shown by OCT imaging (Figure 7a). Additionally, Figure 6b shows the skin surface after microneedle dissolution. This image suggests that curcumin was delivered, as the created pores showed the orange/yellow colour characteristic of curcumin. Moreover, Figure 6c shows the gradual decrease of the needle height as a function of the insertion time. Complete dissolution was observed after 60 min in skin.

**Figure 8:**
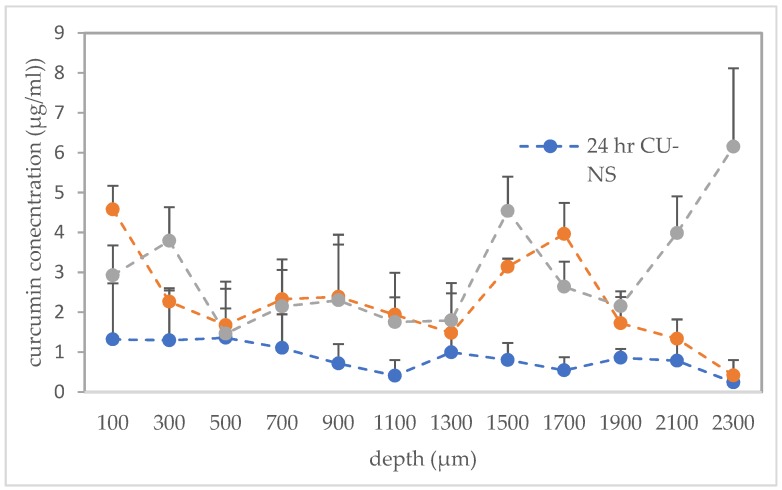
Concentrations of curcumin at different depths in ex vivo neonatal porcine skin for 24 h with CU-MN, for 2 h with CU-MN and for 24 h with the CU-NS alone. Means ± S.D., *n* = 3.

The cryostatic microtome study showed improved localisation of curcumin from the CU-MN array in the deeper skin layers, as compared to the CU-NS alone (Figure 8). Moreover, curcumin levels in the deeper layer (≈ 2300 µm) of the tissue were significantly higher after 24 h of microneedle application compared to the CU-NS and the 2 h CU-MN application (*p* < 0.05 in both cases). There were statistical differences in the total amount of curcumin in skin after 2 h of CU-MN application, 24 h CU-MN application and the 24 h CU-NS application, with the 24 h CU-MN application yielding significantly higher drug deposition than either of the other two and the 2 h CU-MN application depositing significantly more CU than the 24 h CU-NS (*p* = 0.0001 in each comparison).

## 4. Discussion

In our study, several formulations of CU-NS utilising different stabiliser were prepared. Among PVA, SLS, and Tween 80, SLS yielded the lowest particle size and, therefore, was used for subsequent studies. The neutral zeta potential of CU-NS presumably indicates that SLS is not adsorbed on the surface of the nanoparticles. In previous studies, it has been shown that the incorporation of SLS yields smaller particle sizes compared to other stabilisers. For instance, SLS was used previously to prepare zerumbone nanosuspensions, which showed a particle size distribution significantly lower than those obtained using hydroxypropyl methylcellulose (HPMC) as stabiliser [23]. Additionally, Elham et al. [24] prepared glipizide formulations using HPMC, Tween 80, poloxamer and SLS as stabilisers. The only formulations that displayed particle size distributions in the nanoscale range (≈ 260 nm) were those formulated using SLS [24]. In the present formulation, nanoparticles were centrifuged and washed according to a centrifugation/spin cycle to remove any adsorbed SLS from the surface. It has been shown that the zeta potential is typically highly negative in formulations that used SLS as a stabiliser [24]. This is due to adsorption of anionic SLS on the surfaces of the nanoparticles. However, in our formulation, the zeta potential was close to neutral due to the centrifugation/washing steps that presumably resulted in the removal of any adsorbed SLS from the surfaces of the nanoparticles.

It has been demonstrated that the dissolution of curcumin is markedly improved by particle size reduction [25]. In one study, nanosuspensions of curcumin showed a release of 50% at 60 min, compared to 10% using microparticles of curcumin at pH 1.2. At pH 6.8, curcumin nanosuspension release was 95%, compared to 45% for the microparticles at 60 min. [26]. Therefore, in our study, the ability of the of the nanosuspension of curcumin to release faster that curcumin powder was to be expected. Moreover, it is likely that the micron-scale particle size of the unprocessed curcumin powder would yield poor microneedle formulations and varying drug distribution in the microneedle matrices [27]. In contrast, the much smaller CU-NS would be expected to provide better microneedle formulations with uniform drug distributions.

Nanomedicines have been widely incorporated into rapidly dissolving polymeric microneedles for intradermal delivery [28]. To establish the fullest advantage of this delivery system, these microneedles must possess adequate physical strength and skin penetration capabilities. Previously, a wide range of hydrophilic film-forming polymers, including poly(vinylpyrrolidone) (PVP) and PVA, were studied and shown to withstand a 32 N insertion force [10]. This force is comparable to the mean force a human exerts when applying microneedles [19]. The force was applied in our study for 30 s, which is a typical recommended time for microneedle application [29]. The ability of CU-MN to withstand the normal insertion force indicates that the mechanical strength of the microneedles is preserved with the incorporation of the CU-NS in the formulation.

Centrifugation has previously been employed in the preparation of microneedles to concentrate drugs towards the microneedle tips [30,31]. Since microneedles do not typically insert fully into skin, efficiency of delivery can possibly be enhanced by concentrating the drug towards the tips. Enhancing the efficiency of drug delivery could obviously enhance the drug concentrations achievable throughout the skin, as shown in our study.

In order for the incorporated drug to elicit its pharmacological effect, the microneedles must dissolve in the skin and release their cargo. Previously, hydrophilic microneedles composed of PVP and hyaluronic acid were able to dissolve in neonatal porcine skin within 5 min [32]. In our formulation, the incorporation of hydrophobic CU-NS in the formulation possibly delayed the full dissolution of microneedles to 60 min. The nanosuspension clearly also needs to dissolve for the drug to be available to act.

In our study, the significant difference in skin deposition between the CU-MN and the CU-NS is due the fact that nanosuspension particles are too large to permeate through intact *stratum corneum* [33]. However, DMN creates punctures in the skin, allowing the nanosuspension to deposit in the viable skin layers and, in theory, release curcumin over prolonged periods of time. The permeation of the CU-NS control group into different skin layers is presumably due to the dissolution of curcumin at the skin surface and subsequent permeation into different skin layers or, alternatively movement of the particles into skin appendages, followed by dissolution.

The enhanced curcumin deposition from the microneedle formulation into the viable epidermis and dermis compared to control nanosuspension group is substantiated by our previous findings. It is clear that nanosuspension particles can migrate once in the skin, eventually accumulating at the artificial “bottom” of the skin created by the experimental set-up, as seen in the present study. For example, rilpivirine migrated to a depth of 900 µm in excised neonatal porcine skin at 120 min after insertion of a rilpivirine nanosuspension-loaded PVA microneedle array [34]. In contrast, in skin not treated with microneedles, nanoparticles are typically only observed in the hair follicles [35]. The microneedles allow deposition of the nanosuspension in the skin, where it can dissolve over time to provide a therapeutic effect [36,37].

Topical curcumin has been shown to be similar to orally-administered curcumin in suppressing tumour growth in a mouse squamous cell skin cancer model [14]. However, it may be postulated that microneedles can enhance the antitumour activity of curcumin due to enhanced local delivery.

Beside the potential utility of curcumin in the treatment of local diseases, it also has potential in the treatment of systematic diseases, such as various types of cancer and even gastrointestinal tract disorders. Oral bioavailability of curcumin is low [38,39]. Since drug absorption from the rich dermal microcirculation is efficient, microneedles that enhance the delivery efficiency of curcumin could potentially have applications in management of systemic diseases.

## 5. Conclusions

In this work, we showed, for the first time, that a curcumin nanosuspension could be successfully deposited in skin using dissolving microneedle arrays. The vital next steps will be use of a suitable animal model to study in vivo drug disposition and pharmacokinetics to ascertain whether potentially useful systemic curcumin concentrations are achievable, or whether this novel delivery system would be best suited to enhanced local skin delivery. Repeat application studies will also be interesting to confirm that multiple depositions of nanosuspension particles in the same areas of skin are as safe as repeat application of blank microneedles [40].

## Figures and Tables

**Table 1:** Zeta potential of two formulations of curcumin nanosuspension, based on 4 mL sodium laurylsulfate (SLS) and 10 mL SLS, before and after the addition to PVA solutions.

Formula	Zeta Potential (Before Mixing with PVA Solution)	Zeta Potential (After Mixing with PVA Solution)
4 mL SLS	−3.1 ± 2.1	−1.9 ± 2.4
10 mL SLS	−5.4 ± 3.7	−2.3 ± 1.9
